# 72. Evaluation of Acclerate Pheno™ on Clinical and Antimicrobial Outcomes in Patients with Methicillin Susceptible *Staphylococcus aureus* Bloodstream Infections

**DOI:** 10.1093/ofid/ofab466.274

**Published:** 2021-12-04

**Authors:** Deanna Berg, William P DePasquale, Mary L Staicu, Sean Stainton, Mindee Hite, Maryrose R Laguio-Vila

**Affiliations:** 1 Wegmans School of Pharmacy, Rochester Regional Hospital, Penfield, New York; 2 Wegmans School of Pharmacy, Rochester General Hospital, Victor, New York; 3 Rochester General Hospital, Rochester, New York; 4 Rochester Regional Health, Rochester, NY

## Abstract

**Background:**

Anti-staphylococcal beta-lactams (BL) are treatment of choice for methicillin-susceptible *Staphylococcus aureus* (MSSA) bloodstream infections (BSI) as they have superior MSSA bacteremia clearance. Based on the hypothesis that earlier initiation of anti-staphylococcal BL may improve clinical outcomes, this study compared clinical and microbiologic features of patients with MSSA BSI pre- and post-implementation of Accelerate Pheno™ (AXDX).

**Methods:**

This was a case-control analysis of adult inpatients with MSSA BSI analyzed using AXDX compared to traditional laboratory methods. Cases were prospectively identified by the antimicrobial stewardship team between August and October 2020 post implementation of AXDX in July 2020. Patients were matched with historical controls (July 2018–July 2020) based on age (±4 years), gender, organism, and source of infection. The primary outcome was time to antibiotic (abx) deescalation to an anti-staphylococcal beta-lactam. Secondary outcomes included hospital length of stay (LOS), 30-day all-cause mortality and hospital readmission, and 60-day *C. difficile* infection.

**Results:**

A total of 25 cases with MSSA BSI were identified, of which 18 (72%) were matched to historical controls. Of these patients, 12 (67%) were male with an average age of 67 years (SD ±12). Other demographics were similar between groups. The median time to species identification [21.3 hours in cases (IQR 14–31.9) vs 33.3 hours in controls (29–41.7), p=0.046] and abx susceptibilities [22.5 hours (18.8 – 42) vs 60.1 hours (46–61.9), p< 0.001] were significantly shorter in cases. The average time to abx deescalation from time to organism susceptibility was 1.7 days (±1.9) for cases compared to 2.7 days (±1.5) for controls (p = 0.129). There were no significant differences detected in hospital LOS, 30-day mortality or readmission, and 60-day *C.difficile* infection.

**Conclusion:**

Although time to organism identification and abx susceptibilities was significantly shorter in cases, AXDX was not associated with a statistically significant reduction in time to anti-staphylococcal BL initiation nor a difference in associated clinical outcomes. A trend in shorter time to abx de-escalation was observed and warrants further investigation in a larger population.

**Disclosures:**

**All Authors**: No reported disclosures

